# The Effect of Temperature on the Mechanical Properties of Alginate Gels in Water/Alcohol Solutions

**DOI:** 10.3390/gels9070579

**Published:** 2023-07-16

**Authors:** Haniyeh Malektaj, Aleksey D. Drozdov, Jesper deClaville Christiansen

**Affiliations:** Department of Materials and Production, Aalborg University, Fibigerstraede 16, 9220 Aalborg, Denmark; aleksey@mp.aau.dk (A.D.D.); jc@mp.aau.dk (J.d.C.)

**Keywords:** alginate, organohydrogel, antifreeze, alcohol

## Abstract

Alginate organohydrogels prepared in water/alcohol mixtures play an important role in electronic and superconductor applications in low-temperature environments. The study deals with the preparation of Ca-alginate organohydrogels and the analysis of their equilibrium swelling and mechanical properties at sub-zero temperatures. It is shown that the equilibrium degree of swelling at room temperature is noticeably affected by the concentration of co-solvents (methanol, ethanol, and 2-propanol) in the mixtures and the number of carbon atoms in the co-solvent molecules. Mechanical properties are studied in small-amplitude oscillatory tests. The data are fitted with a model that involves three material parameters. The influence of temperature is investigated in temperature-sweep oscillatory tests under a cooling-heating program, where a noticeable difference is observed between the storage and loss moduli under cooling and heating (the hysteresis curves). The hysteresis areas are affected by the cooling/heating rate and the number of carbon atoms in the co-solvents.

## 1. Introduction

Lithium-ion batteries are a promising technology for energy storage because of their high energy and power density, nearly zero memory effect, low self-discharge property, high open circuit voltage, and long service life [1]. Applications of these batteries are limited, however, by their poor performance at low temperatures [2,3]. The cell capacity, working temperature range, safety issues, and cyclability of lithium-ion batteries are affected by the selected electrolyte [4,5]. Liquid electrolytes have high ionic conductivity, although safety problems, leakage of electrolytes, undesired dislocation under strain, and the side effects of oxygen and water on electrode reactions have challenged the practical applications of liquid electrolytes [4]. The development of solid electrolytes faces challenges. Large-scale fabrication of solid electrolytes is difficult due to their fragility, dissatisfactory contact between solid electrolytes and electrodes, and dendrite growth along grain boundaries inside the solid electrolytes. An additional challenge is that solid electrolytes need to be mixed with active materials to facilitate ionic transport [6]. Hydrogel electrolytes seem to be a more promising option for high-performance lithium-ion batteries due to their safety, mechanical flexibility, and electrode–electrolyte interfacial stability [7]. Hydrogel electrolytes can maintain their physical integrity and flexibility under various mechanical deformations [8], have a limited free water content, and suppress the reactions of water in the batteries [9,10].

Hydrogel electrolytes are made from polymers such as poly (ethylene oxide) (PEO) [11], polyvinyl alcohol (PVA) [8], polyacrylamide (PAM) [12], xanthan gum [13], cellulose, chitosan, and alginate [14]. Alginate gels cross-linked by calcium ions can be used as an electrolyte [14], and the presence of the -COOH group in their structure leads to the ability to interact with salt anions, thereby increasing cation transport properties [15].

The benefits that most conventional gel electrolytes reveal under mild environmental conditions disappear, however, at subzero temperatures due to the relatively high freezing point of water [13]. This can dramatically decrease the mechanical properties and ionic conductivity of these hydrogels. A commonly reported strategy for preparing antifreeze hydrogels is to replace water with organic solvents. This lowers the freezing point of the gels and prevents the formation of hydrogen bonds between polymer chains and water [16]. Among organic solvents, alcohol compounds are widely used in industry because of their low cost and easy production. Most alcohols exhibit high water solubility due to the presence of hydroxyl groups [17]. A double-network organohydrogel of polyacrylamide and sodium alginate cross-linked with calcium chloride (CaCl_2_) was used at temperatures down to −20 °C [18]. An alginate-based organohydrogel was prepared in [19], where its antifreeze performance below −42.1 °C was demonstrated. A sodium-alginate hydrogel was manufactured by partial solvent replacement (with glycerol and PEG) in [20], where its anti-freezing and heat-resistant properties were revealed at a wide range of temperatures between −70 °C and 120 °C. Hu et al. [21] synthesized a PVA/alginate organohydrogel by incorporation of glycerol and showed its high transparency, anti-freezing, and thermoplastic properties.

In addition to their antifreeze properties, gels with tunable mechanical properties are attractive for their use as flexible electrolytes or supercapacitor electrodes. The PVA-based organohydrogel remained flexible and could be twisted after being stored at −18 °C. Loading-unloading tests under different strains indicated that PVA-based organohydrogels could dissipate energy, and the hysteresis energy increased with the rise of strain [22]. A tough anti-freezing Ca-alginate/polyacrylamide organohydrogel was fabricated with glycerol with a wide operating temperature range down to −70 °C [23]. However, more investigations are required to characterize the effect of temperature on the mechanical properties and energy dissipation of organohydrogels.

Besides the anti-freezing ability, the hydrogen bonds formed between alcohol and water molecules ensure the anti-drying ability of organohydrogels [24,25]. Although water causes a gel to freeze, its optimal amount and moisture retention combined with anti-freezing capacities result in superior stability of gel-based electrical devices. Specific salts (such as CaCl_2_) incorporated into gels provide better moisture retention capacity. Ion-conducting PAM/calcium alginate organohydrogel showed long-term stability together with anti-drying and anti-freezing abilities [26]. Moreover, in [27], the ionic compound potassium iodide (KI) was introduced into the glycerol-based PAAm organogel to achieve a conductive gel sensor that could be used in the temperature range between −30 °C and 60 °C. The conductivity of organohydrogels is related to ion transportation and mobility [28]. The swelling behavior of organohydrogels is crucial in controlling ion diffusion and mobility. An optimal swelling ability ensures structural integrity and sufficient mechanical support, even during the freezing processes. Although the degree of swelling should be controlled, gels with anti-swelling properties are suitable to prevent damage of the gel electrolytes during operational conditions. An organohydrogel with ionic conductivity and anti-swelling properties was prepared in [29].

Hydrogels with a high density of covalent cross-links slow down ion transport, creating a dilemma between strength and ionic conductivity [30]. Due to this shortcoming, we focus on the analysis of ionically cross-linked organohydrogels to be used as gel electrolytes. In the previous paper [31], we prepared uniform alginate hydrogels cross-linked with Ca^2+^ ions. The swelling properties of these gels were studied in aqueous solutions with various pH values and ionic strengths. This work analyzes the equilibrium swelling properties of ionically cross-linked alginate gels soaked in organic solvents (methanol, ethanol, and 2-propanol) at various temperatures. The temperature dependence of the elastic modulus of alginate gels at elevated temperatures was first demonstrated in [32]. This study investigates the influence of temperature on the storage and loss moduli of alginate organohydrogels at negative temperatures down to −50 °C.

The objective of this study is twofold.

Our first aim was to prepare anti-freezing organohydrogels and study the effect of temperature on their equilibrium degree of swelling. After the preparation of Ca-alginate hydrogels, they were immersed in aqueous solutions of methanol, ethanol, and 2-propanol with various volume fractions of co-solvents (ranging from 25 to 100 wt.%). We determined the diffusivity *D* of water/alcohol mixtures at low temperatures and found that equilibrium swelling could not be reached at low temperatures within a reasonable experimental time. Instead, we used dynamic mechanical thermal analysis (DMTA) to characterize the mechanical properties of organohydrogels at sub-zero temperatures.

The other aim was to investigate the effect of temperatures and frequency on the storage and loss moduli of alginate organohydrogels in water/alcohol mixtures. For this purpose, these organohydrogels (immersed in aqueous solutions with 80 wt.% co-solvents) were tested with DMTA at temperatures *T* ranging from 22 °C down to −50 °C and frequencies belonging to the interval between 0.1 and 80 Hz. It was shown that the chemical structure and concentration of co-solvents in the mixtures affected noticeably the viscoelastic properties of organohydrogels.

This study focuses on the evaluation of the mechanical and swelling properties of ionically cross-linked alginate organohydrogels. An analysis of the ionic transport in these gels under an electric field will be a subject of the subsequent publication.

## 2. Results and Discussion

### 2.1. Results

#### 2.1.1. Swelling of Alginate Hydrogels in Solutions with Various Volume Fraction of Alcohols

**Figure 1:**
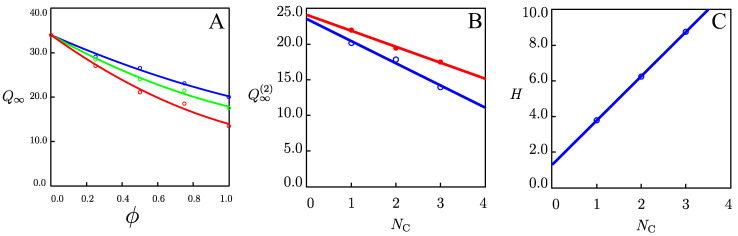
Equilibrium degree of swelling $Q_{\infty }$ versus volume fraction of co-solvent ϕ. Circles: experimental data in swelling tests on alginate organohydrogel in mixtures of water with methanol (blue), ethanol (green), and 2-propanol (red) at temperature *T* = 22 °C (**A**). Parameters $Q_{\infty }^{\left(2\right)}$ (**B**) and H (**C**) versus the number of carbon atoms $N_{C}$ in co-solvent molecules. Unfilled circles: treatment of observations in equilibrium swelling tests in pure alcohols at temperature *T* = 22 °C. Filled red circles: experimental data after a week of swelling of alginate organohydrogel in pure alcohols at temperature *T* = −20 °C. Solid lines: results of numerical analysis.

To assess the effect of the concentration of alcohols in water/alcohol mixtures on the swelling of organohydrogels, their equilibrium degree of swelling $Q_{\infty }$ (Equation (9)) was measured in aqueous solutions with methanol, ethanol, and 2-propanol with volume fractions ϕ of 25, 50, 75, and 100 wt.% at temperature *T* = 22 °C. The results are presented in Figure 1A, where the equilibrium degree of swelling $Q_{\infty }$ was plotted as a function of volume fractions ϕ of alcohols. The equilibrium degree of swelling $Q_{\infty }$ decreases with the volume fraction ϕ of alcohols. The dependence of $Q_{\infty }$ on ϕ was approximated by the Equation [33].

(1) $$Q_{\infty }=Q_{\infty }^{\left(1\right)}\left(1-\phi \right)+Q_{\infty }^{\left(2\right)}\phi -H\phi \left(1-\phi \right)$$

Equation (1) is typical for the treatment of experimental data on composites. Here $Q_{\infty }^{\left(1\right)}$, and $Q_{\infty }^{\left(2\right)}$ stand for the equilibriums degree of swelling of organohydrogels in pure water, and pure alcohols, respectively. The parameter *H* characterizes the effect of mixing of water with alcohol on the equilibrium degree of swelling $Q_{\infty }$. The coefficients $Q_{\infty }^{\left(2\right)}$ and *H* were found by the nonlinear regression and were plotted as functions of the number of carbon atoms $N_{C}$ in alcohol molecules in Figure 1B,C, where they were approximated by the linear dependencies. In our analysis, H serves as a measure of the hydrophobicity of the mixtures [34]. Figure 1C shows that when the number of carbon atoms $N_{C}$ in alcohol molecules increases, the hydrophobicity of the mixture grows. Figure 1B shows that the $Q_{\infty }^{\left(2\right)}$ parameter decreases with hydrophobicity.

To assess the diffusivity *D* of the mixtures at low temperatures *T*, we perform swelling experiments at high temperatures *T* (results are presented in Figure S1). The coefficient of diffusion *D* was calculated by fitting the initial intervals of the experimental dependencies of the degree of swelling *Q* on time *t* using Equation (2) [35,36]

(2) $$\frac{Q}{Q_{\infty }}=\frac{4}{a}{\left(\frac{Dt}{\pi }\right)}^{\frac{1}{2}}$$

where *a* denotes the height of a disk sample.

**Figure 2:**
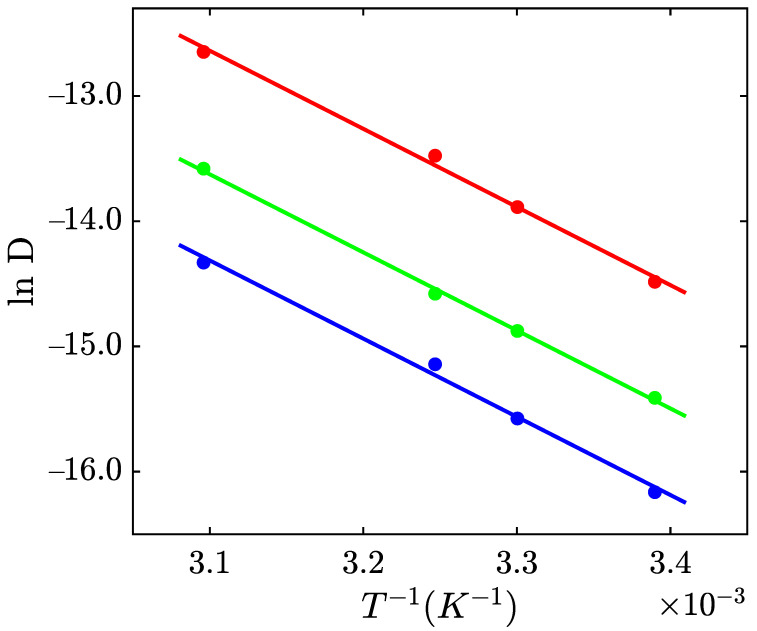
Cofficient *D* versus temperature *T*. Circles: experimental data on the organohydrogel in pure methanol (blue), ethanol (green), and 2-propanol (red).

Afterward, we approximate coefficients *D* as a function of temperature *T* by the Arrhenius equation.

(3) $$D=D_{0}exp\left(-\frac{E_{a}}{RT}\right)$$

where $D_{0}$ stands for a pre-factor, $E_{a}$ is the activation energy for diffusion of solvent, *R* is the universal gas constant, and *T* denotes the absolute temperature. The experimental data and their fits by Equation (3) are reported in Figure 2.

We calculated the coefficient *D* at temperatures *T* = −20 °C and −50 °C by means of Equation (3) and found that the times necessary to reach the equilibrium degree of swelling of gel samples in pure 2-propanol, ethanol, and methanol are around 20, 60, 90 days, and 1.25, 4.5, 6.55 years at temperatures *T* = −20 °C and −50 °C, respectively.

For comparison, the degree of swelling *Q* of the organohydrogels after one week of swelling in pure alcohols at temperature *T* = −20 °C is presented in Figure 1B (filled circles). As the diffusivity *D* at temperature *T* = −20 °C is extremely low, Figure 1B shows that our data after one week of swelling differ from the corresponding equilibrium degree of swelling $Q_{\infty }^{\left(2\right)}$.

#### 2.1.2. The Effects of Co-Solvents on Mechanical Properties of Organohydrogels

**Figure 3:**
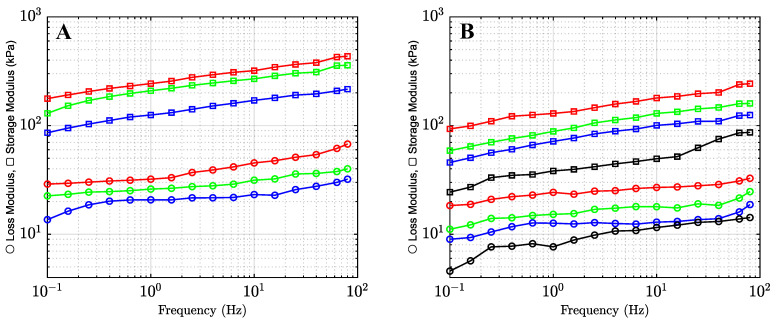
Storage modulus $E^{\prime }$ and loss modulus $E^{\prime \prime }$ versus frequency *f* for the organohydrogels in mixtures of water with 80% (*v*/*v*) of methanol (blue), ethanol (green), 2-propanol (red), and water with pH = 7 (black) at −40 °C (**A**) and 22 °C (**B**).

To study the mechanical properties of alginate organohydrogels at sub-zero temperatures, we perform small-amplitude oscillatory tests in the frequency mode (materials and methods section) at temperature *T* = −40 °C. This temperature was chosen because it is considered the temperature limit for most battery applications [37]. The logarithms of the storage modulus $E^{\prime }$ and the loss modulus $E^{\prime \prime }$ are depicted versus the logarithm of frequency *f* at temperature *T* = −40 °C in Figure 3A. For comparison, observations on the storage and loss moduli at temperature *T* = 22 °C are presented in Figure 3B.

Observations show that the storage and loss moduli at *T* = −40 °C are higher than those at 22 °C. An increase in stiffness of the organohydrogels is driven by the formation of clusters between water and alcohol molecules at sub-zero temperatures [38]. See the Discussion section.

We fitted the experimental data in Figure 3A,B using the model proposed in [39,40]. The storage, $E^{\prime }(\omega )$, and loss, $E^{\prime \prime }(\omega )$, moduli are given by.

(4) $$E^{\prime }(\omega )=E\int_{0}^{\infty }f(v)\frac{\left(1-\kappa \right)\;\Gamma^{2}\left(v\right){+\omega }^{2}}{\Gamma^{2}\left(v\right)+\omega^{2}}dv$$

(5) $$E^{\prime \prime }\left(\omega \right)=E\int_{0}^{\infty }f(v)\frac{\kappa \;\Gamma \left(v\right)\omega }{\Gamma^{2}\left(v\right)+\omega^{2}}dv$$

The rate of thermally induced breakage of reversible bonds (ionic and hydrogen bonds) between chains 𝛤(𝑣) is determined by the Eyring formula.

(6) $$\Gamma \left(v\right)=\Gamma_{0}exp(-v)$$

where v is a dimensionless activation energy, and $\Gamma_{0}$ is a pre-factor.

The inhomogeneity of a polymer network is characterized by the probability density f(v) to find a reversible bond with an activation energy v. The latter is described by the quasi-Gaussian formula.

(7) $$f\left(v\right)=f_{0}\exp\left(-\frac{v^{2}}{2\;\Sigma^{2}}\right),$$

where Σ  is a measure of inhomogeneity.

According to the model, a temporary polymer network in a gel consists of flexible chains connected by reversible bonds that break and reform at random instants, driven by thermal fluctuations. Each bond is characterized by its activation energy v. The distribution of bonds with various activation energies for breakage is determined by the function f(v) that characterizes the inhomogeneity of the network. After breakage of a temporary bond, stresses in chains linked by this bond vanish. When a new temporary bond is formed, the initial (reference) state of a chain merged by this bond coincides with the current state of the network [40].

With reference to the “egg-box” model for alginate gels cross-linked with divalent cations [41], we suppose that the inhomogeneity of the polymer network in these gels is mainly driven by the number of cations and G-blocks forming a particular zipping structure (which is treated as a transient bond) between two nearby chains.

Given an angular frequency ω=2πf, Equations (4) and (5), together with Equation (6), with $\Gamma_{0}$ = 15.000 s^−1^ and Equation (7) for $f\left(v\right)$ involve three material parameters: (i) *E* stands for the elastic modulus of a gel, (ii) Σ is a measure of its inhomogeneity, and (iii) κ is the concentration of reversible bonds between chains.

**Figure 4:**
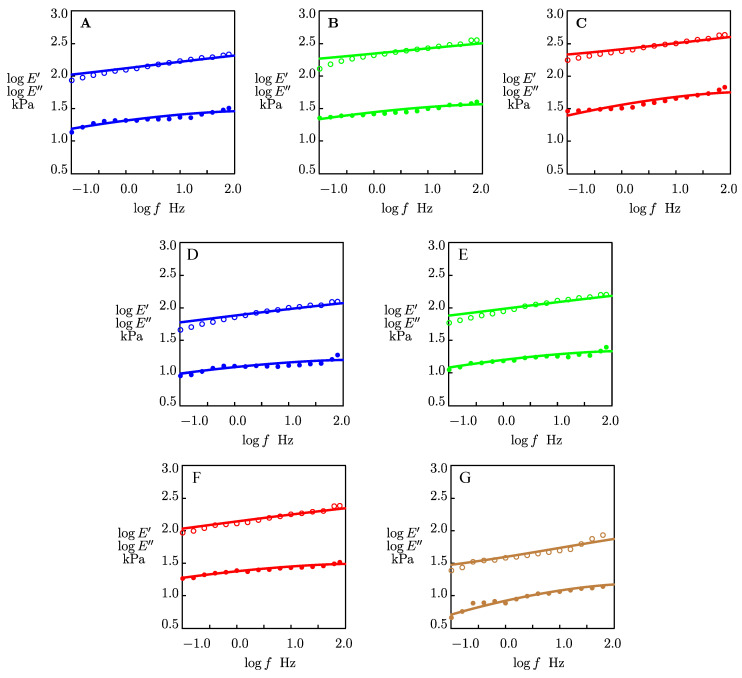
The storage $E^{\prime }$ (◦) and loss $E^{\prime \prime }$ moduli (•) versus frequency *f*. Symbols: experimental data in small-amplitude oscillatory tests on alginate gel in water/methanol (**A**), water/ethanol (**B**), water/2-propanol (**C**) mixtures at temperature *T* = −40 °C, and in water/methanol (**D**), water/ethanol (**E**), and water/2-propanol (**F**) mixtures, and pure water with pH = 7 (**G**) at temperature *T* = 22 °C. Solid lines: results of numerical analysis.

**Figure 5:**
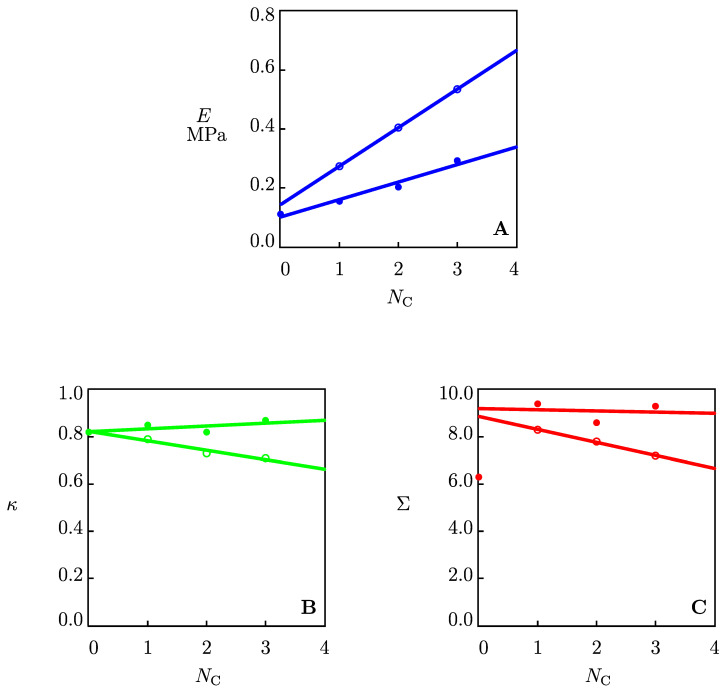
The elastic modulus *E* (**A**) and the parameters κ (**B**) and Σ (**C**) versus the number of carbon atoms $N_{C}$ in co-solvent molecules. Symbols: treatment of experimental data at temperatures *T* = −40 (◦) and *T* = 22 (•) °C. Solid lines: results of numerical simulation.

These coefficients are found by fitting the experimental data in Figure 4. This figure shows an acceptable agreement between the data and the results of the numerical simulation. The parameters *E*, κ and Σ are plotted as functions of the number of carbon atoms in alcohol molecules $N_{C}$ in Figure 5. The data were approximated by the linear functions. Figure 5A shows an increase in the elastic modulus *E* with the number of carbon atoms $N_{C}$. Introducing co-solvents with a higher number of carbon atoms $N_{C}$ into the gel network enhances the elastic modulus of organohydrogels. The effect of $N_{C}$ on the parameters κ and Σ is illustrated in Figure 5B,C. These figures show that co-solvents have no significant effect on *κ* and Σ at temperature *T* = 22 °C, while κ and Σ parameters decrease with the $N_{C}$ at temperature *T* = −40 °C.

The growth of the elastic modulus *E* with the hydrophobicity of the co-solvent (characterized by the parameter $N_{C}$) may be explained by interactions between water and co-solvent molecules. As a result of these interactions, water molecules forming hydrogen bonds with alginate chains (the bound water) leave the chains. This leads to the formation of new hydrogen bonds between surrounding chains. The higher the number of water molecules that break their hydrogen bonds with alginate chains (to form new bonds with co-solvent molecules), the higher the number of extra hydrogen bonds formed between chains, and the larger the elastic modulus of the polymer network (Figure 5A). Figure 5B,C reveal that the coefficients *κ* and Σ are practically unaffected by $N_{C}$ at room temperature, which means that new hydrogen bonds between alginate chains do not affect the structure of “egg-boxes” that form reversible bonds between chains. On the contrary, changes in *κ* and Σ with $N_{C}$ at *T* = −40 °C indicate that new hydrogen bonds between chains affect the zipping structure of an alginate gel, which may be attributed to changes in affinity between G-blocks and divalent cations at low temperatures.

**Figure 6:**
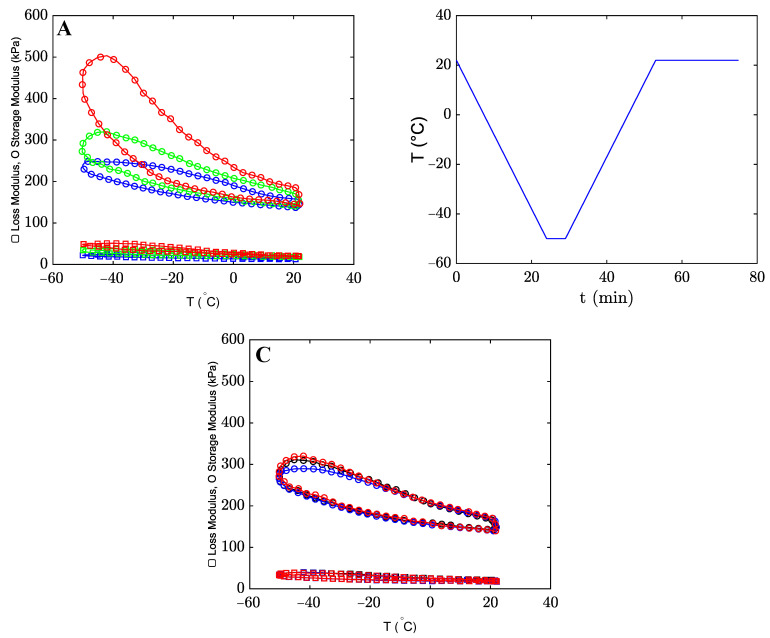
Storage and loss moduli versus temperature *T* for the organohydrogels in mixtures of water with 80% (*v*/*v*) methanol (blue), ethanol (green), and 2-propanol (red) with a 3 °C/min cooling/heating rate (frequency = 10 Hz, strain amplitude = 0.5%) (**A**). Thermo-program of the hysteresis curve (**B**). Three repetitions on the same sample in a mixture of water with 80% (*v*/*v*) ethanol (**C**).

To evaluate the effect of temperature *T* on the mechanical properties, the oscillatory tests were performed when temperature *T* decreased from 22 °C to −50 °C and increased back to 22 °C (Section 4). The effect of temperature *T* on the loss and storage moduli is shown in Figure 6A. The thermal program for a cooling/heating cycle (with a 3 °C/min rate) is illustrated in Figure 6B.

The cooling-heating hysteresis curves are typical for the organohydrogels under consideration. Under cooling, the loss and storage moduli of the organohydrogels increase for all co-solvents. We hypothesize that water in the organohydrogels forms clusters with alcohols at subzero temperatures, and this leads to an increase in the stiffness of the polymer network. The growth of the storage modulus occurs more pronouncedly in co-solvents with a higher number of carbon atoms $N_{C}$. Upon heating, the bonds between water and alcohols weaken, and the organohydrogel recovers its original structure with the initial loss and storage moduli at temperature *T* = 22 °C.

Figure 6C shows that the hysteresis curves for the organohydrogel coincide practically for all cycles, which indicates that the network structure of the organohydrogel does not experience permanent changes along each cycle.

**Figure 7:**
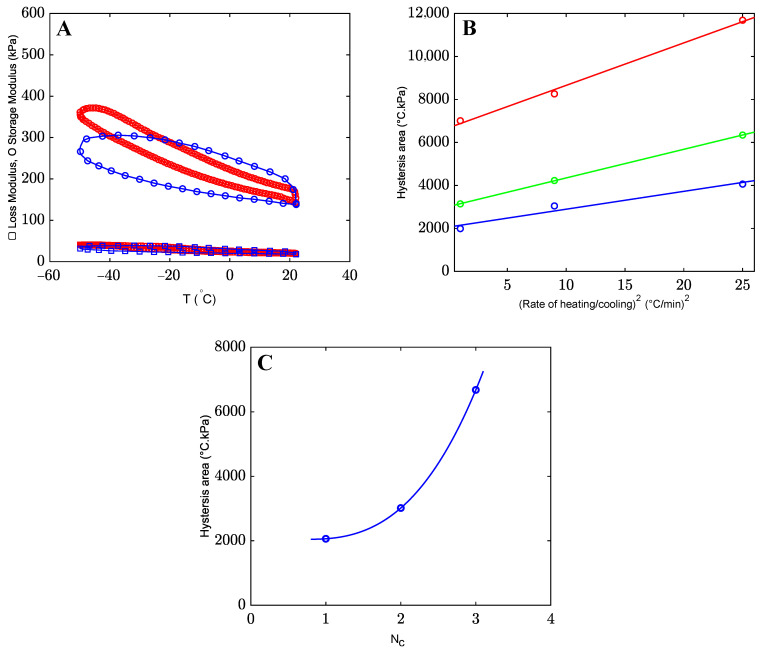
Storage and loss moduli versus temperature *T* for the organohydrogels in mixture of water with 80% (*v*/*v*) ethanol with 1 °C/min (red) and 5 °C/min (blue) cooling/heating rate (frequency = 10 Hz, strain amplitude = 0.5%) (**A**). The hysteresis area for the organohydrogels in mixtures of water with 80% (*v*/*v*) methanol (blue), ethanol (green), and 2-propanol (red) versus cooling/heating rate (**B**). The hysteresis area at zero cooling/heating rate versus the number of carbon atoms $N_{C}$ in co-solvent molecules (**C**).

The same tests with 1 and 5 °C/min cooling/heating rates were performed to evaluate the effect of the cooling/heating rates on the hysteresis curve. Figure 7A shows that the loss and storage moduli increase further as the cooling/heating rate decreases. In [42], it was assumed that water/alcohol clusters need a fixed period of time to approach a steady state. Figure 7A shows that the clusters do not reach a stable state at relatively high cooling rates. When the cooling rate decreases, larger and more stable clusters are formed, resulting in an increase in the storage modulus. During the heating process, energy is dissipated as the bonds between water and alcohol gradually weaken. Figure 7B shows the area of the hysteresis loop (which serves as a measure of dissipated energy [19]) as a function of the heating/cooling rate. The dissipated energy grows with the cooling/heating rate. Figure 7C demonstrates an increase in the hysteresis area when organohydrogels are immersed in the co-solvent with a higher number of carbon atoms *N_C_*.

### 2.2. Discussion

Anti-freezing alginate organohydrogels have been prepared by immersion of alginate hydrogel into water/methanol, ethanol, and 2-propanol mixtures, and their mechanical and swelling properties have been investigated experimentally.

The equilibrium degree of swelling $Q_{\infty }$ in water/alcohol mixtures decreases with volume fractions ϕ of alcohols at temperature *T* = 22 °C. The swelling data are fitted by Equation (1) with two coefficients, $Q_{\infty }^{\left(2\right)}$ and *H*. The parameter *H* increases linearly with *N_C_*, which serves as a measure of the hydrophobicity of mixtures [34]. The equilibrium degree of swelling in pure alcohols $Q_{\infty }^{\left(2\right)}$ decreased with an increase in this measure of hydrophobicity.

Based on the calculation of diffusivity *D*, it is found that the times necessary to reach the equilibrium degree of swelling $Q_{\infty }$ in pure 2-propanol, ethanol, and methanol are around 20, 60, 90 days, and 1.25, 4.5, and 6.55 years at temperatures *T* = −20 °C and −50 °C, respectively. To avoid long-term experiments, the mechanical properties of the organohydrogel at sub-zero temperatures were determined by means of DMTA.

The small-amplitude oscillatory tests in the frequency-sweep mode show that the storage modulus is higher than the loss modulus throughout the frequency range (0.1–80 Hz) at temperatures *T* = 22 and −40 °C, indicating the dominant elastic behavior [43]. This means that the alginate organohydrogels are mechanically stable under compression [44]. The experimental data in small-amplitude oscillatory tests are fitted with a model with only three adjustable parameters. It is found that the elastic modulus *E* of organohydrogels increased with the hydrophobicity of the co-solvent at both temperatures *T* = 22 and −40 °C. The inhomogeneity of the polymer network Σ, and the concentration of reversible bonds between chains *κ* decreased consistently with $N_{C}$ at *T* = −40 °C and remained practically independent of $N_{C}$ at *T* = 22 °C.

In oscillatory tests with a constant frequency of 10Hz, the loss and storage moduli of the organohydrogels increase when the temperature decreases from 22 to −50 °C. The growth of the storage and loss moduli does not occur due to the formation of new ionic bonds between alginate chains [32]. When the temperature decreases, more and more clusters formed by water and alcohol molecules are formed [45]. As a result, water molecules that form hydrogen bonds with polymer chains (bound water) leave the network. Due to this process, new hydrogen bonds are formed between surrounding alginate chains, which leads to stiffening of the organohydrogels. The size of clusters formed by water and alcohol molecules depends on the type and concentration of alcohols [46]. In co-solvents with high numbers of carbon atoms $N_{C}$ (and higher hydrophobicity), larger clusters are formed, which requires larger amounts of water molecules (bound water molecules) to leave the chains. As a result, more hydrogen bonds arise between nearby chains, which causes a further increase in the storage and loss moduli. The rate of formation of water/alcohol clusters under cooling is affected by the rate of decrease in temperature. At lower cooling rates, more clusters are formed, which leads to a more pronounced increase in the elastic modulus *E*. Under heating (from −50 to 22 °C), these clusters dissociate, and free water molecules return to the polymer network by forming hydrogen bonds between chains. As a result, part of the hydrogen bonds between surrounding chains break, its modulus decreases, and the organohydrogel recovers its initial structure at room temperature, where alginate chains are linked by both ionic and hydrogen bonds [47]. This picture is confirmed by the observations depicted in Figure 7B, which show that more energy is dissipated under hysteresis with higher cooling/heating rates, as water–alcohol clusters do not have enough time to break under heating.

This study demonstrates that alginate organohydrogels prepared by the proposed method have anti-freezing properties up to −50 °C and can be used in electronic and superconductor applications. Our strategy is to depress the freezing point of hydrogels by introducing alcohols into the hydrogel. When a sample is placed in a water/alcohol mixture, the quantity of free water in the gel decreases [48]. At low temperatures, water forms clusters with alcohols, which implies that the organohydrogels remain unfrozen. The equilibrium degree of swelling $Q_{\infty }$ and mechanical properties of the alginate organohydrogels are affected by the hydrophobicity of water/alcohol mixtures.

## 3. Conclusions

Alginate organohydrogels were prepared in water/alcohol mixtures, and the effects of temperature and co-solvent on their swelling and mechanical properties were studied. It is found that the presence of alcohols in the organohydrogels prevents the formation of hydrogen bonds between water molecules and makes the organohydrogels resistant to freezing. In swelling tests, we observed that the equilibrium degree of swelling $Q_{\infty }$ decreased with an increase in the number of carbon atoms $N_{C}$ in co-solvent molecules at temperature *T* = 22 °C. The swelling data were fitted by a model with two parameters. It is found that the equilibrium degree of swelling in pure alcohols $Q_{\infty }^{\left(2\right)}$ and the coefficient *H* in Equation (1) change linearly with $N_{C}$. Due to the low diffusivity at subzero temperatures, equilibrium cannot be reached within reasonable time intervals, and DMTA analysis was used to measure the mechanical properties of the organohydrogels. The experimental data in small-amplitude oscillatory tests were fitted by a model that involves three adjustable parameters. It was found that the elastic modulus *E*, the inhomogeneity of the polymer network Σ, and the concentration of reversible bonds between chains *κ* change consistently with $N_{C}$ at *T* = −40 °C. It was revealed that alginate organohydrogels could recover their structure after cooling and subsequent heating. The hysteresis curves show that the dissipated energy decreases when the cooling/heating rates decrease. This study shows that alginate organohydrogels have acceptable antifreeze and mechanical properties at temperatures down to −50 °C and can be used in electronic and superconductor applications. An analysis of the ionic transport in alginate organohydrogels under an electric field will be conducted in a subsequent publication.

## 4. Materials and Methods

### 4.1. Materials

Alginic acid sodium salt from brown algae was purchased from Acros Organics (Geel, Belgium). CaCl_2_ and potassium hydroxide (KOH) were provided by Sigma–Aldrich. Hydrochloric acid (HCl), methanol, ethanol, and 2-propanol were supplied by VWR International (Rosny-sous-Bois, France). Deionized water was used in hydrogel preparation and measurements.

### 4.2. Preparation of Organohydrogels

Alginate hydrogels were prepared by a method described previously in [31]. Briefly, 1 wt.% alginate solution (pH was adjusted to 3.5 by adding HCl) was mixed with 50 mM of CaCl_2_ solution in a proportion of 28:1 (*v*/*v*), and the mixture was poured into a mold and kept overnight. The weak hydrogel was crosslinked by immersing it in a strong CaCl_2_ solution (1 M) for 2 days to finalize the cross-linking process. Finally, the hydrogel was immersed into a water/alcohol solution (methanol, ethanol, and 2-propanol with various volume fractions ranging from 25 to 100 wt.%) for one week to prepare an organohydrogels.

**Figure 8:**
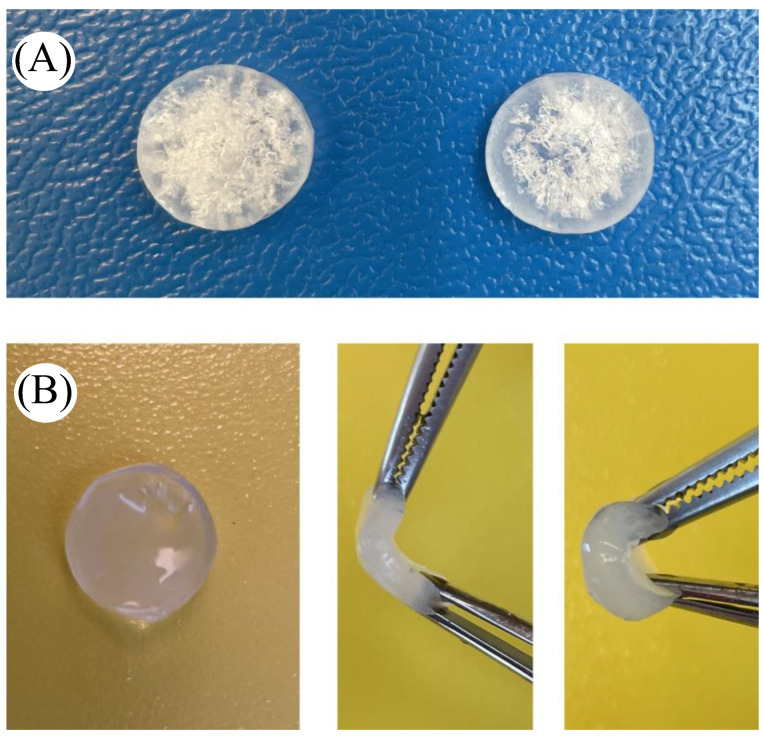
Freezing of alginate hydrogels (**A**), and anti-freezing of alginate organohydrogels at *T* = −20 °C (**B**).

To confirm the antifreeze properties of the alginate organohydrogel, we stored alginate hydrogel and organohydrogel samples at *T* = −20 °C for one week. Figure 8 shows that the alginate organohydrogel keeps its structure after storage at *T* = −20 °C (Figure 8b), while the structure of the alginate hydrogel was destroyed by ice at *T* = −20 °C (Figure 8a).

### 4.3. Swelling Tests

Swelling tests were conducted on organohydrogels immersed in water/alcohol mixtures with various volume fractions of alcohols ranging from 25 to 100 wt.% at temperatures *T* = −20 °C and 22 °C. We used disc-shaped samples with a diameter of 8.5 mm and a height of 2.5 mm. Samples in water/alcohol mixtures at temperature *T* = 22 °C reached equilibrium after one week of swelling. It was shown in [31] that one week is sufficient for its equilibration. We expect that the organohydrogel will not reach equilibrium after one week of swelling at temperature *T* = −20 °C. Thus, we stopped the swelling test at temperature *T* = −20 °C after one week and measured the degree of swelling *Q* at this instant.

The degree of swelling *Q* and the equilibrium degree of swelling $Q_{\infty }$ were determined by the formulas:

(8) $$Q=\frac{w_{t}-w_{0}}{w_{0}}$$

(9) $$Q_{\infty }=\frac{w_{\infty }-w_{0}}{w_{0}}$$

where $w_{0}$, $w_{t}$ and $w_{\infty }$ stand for the initial weight of a sample under preparation, the weight of a sample at time *t*, and the equilibrium weight, respectively.

Five repetitions for all measurements were made on different samples prepared by the same procedure. Experimental data are presented as mean values. The scatter of data is relatively small. The standard deviations of observations in all tests were below 5% of their mean values.

### 4.4. Mechanical Tests

The storage modulus and the loss modulus were measured by means of DMTA Q800 V20.9 in compression oscillatory tests (the temperature-sweep mode) with a strain amplitude of 0.5% and temperature *T* ranging from 22 °C to −50 °C and then back to 22 °C. The cooling/heating rates were 1, 3, and 5 °C/min, and the frequency *f* was 10 Hz.

The small-amplitude oscillatory test in frequency mode was performed at temperatures *T* = −40 °C and 22 °C. Each specimen was thermally equilibrated for 20 min before measurements. The storage and loss moduli were measured at various frequencies *f* starting from 0.1 to 80 Hz.

Three repetitions of measurements were made. Each test was performed on a new sample. We used disc-shaped samples with a diameter of 8.5 mm and a height of 4.5 mm immersed in mixtures of water with 80 wt.% of co-solvent. The standard deviations of the observations do not exceed 5% of their mean values.

## Data Availability

The data that support the findings of this study are available from the corresponding author, H.M., upon reasonable request.

## Supplementary Materials

The following supporting information can be downloaded at: https://www.mdpi.com/article/10.3390/gels9070579/s1, Figure S1: Degree of swelling *Q* versus time *t* for organohydrogels in pure (A) methanol, (B) ethanol and (C) 2-propanol at various temperatures *T*.
