# Supplementary material for: The Effect of Temperature on the Mechanical Properties of Alginate Gels in Water/Alcohol Solutions
Source: Gels. 2023 Jul 16;9(7):579. doi: 10.3390/gels9070579 (PMC10378887; doi:10.3390/gels9070579)
Supplement: Supplementary file 1 [file gels-09-00579-s001.zip › gels-2488871-supplementary.pdf]

Supplementary material

The effect of temperature on the mechanical properties of alginate gels in water/alcohol solutions

H. Malektaj, A.D. Drozdov, J. deClaville Christiansen

Department of Materials and Production

Aalborg University

Fibigerstraede 16, Aalborg 9220, Denmark

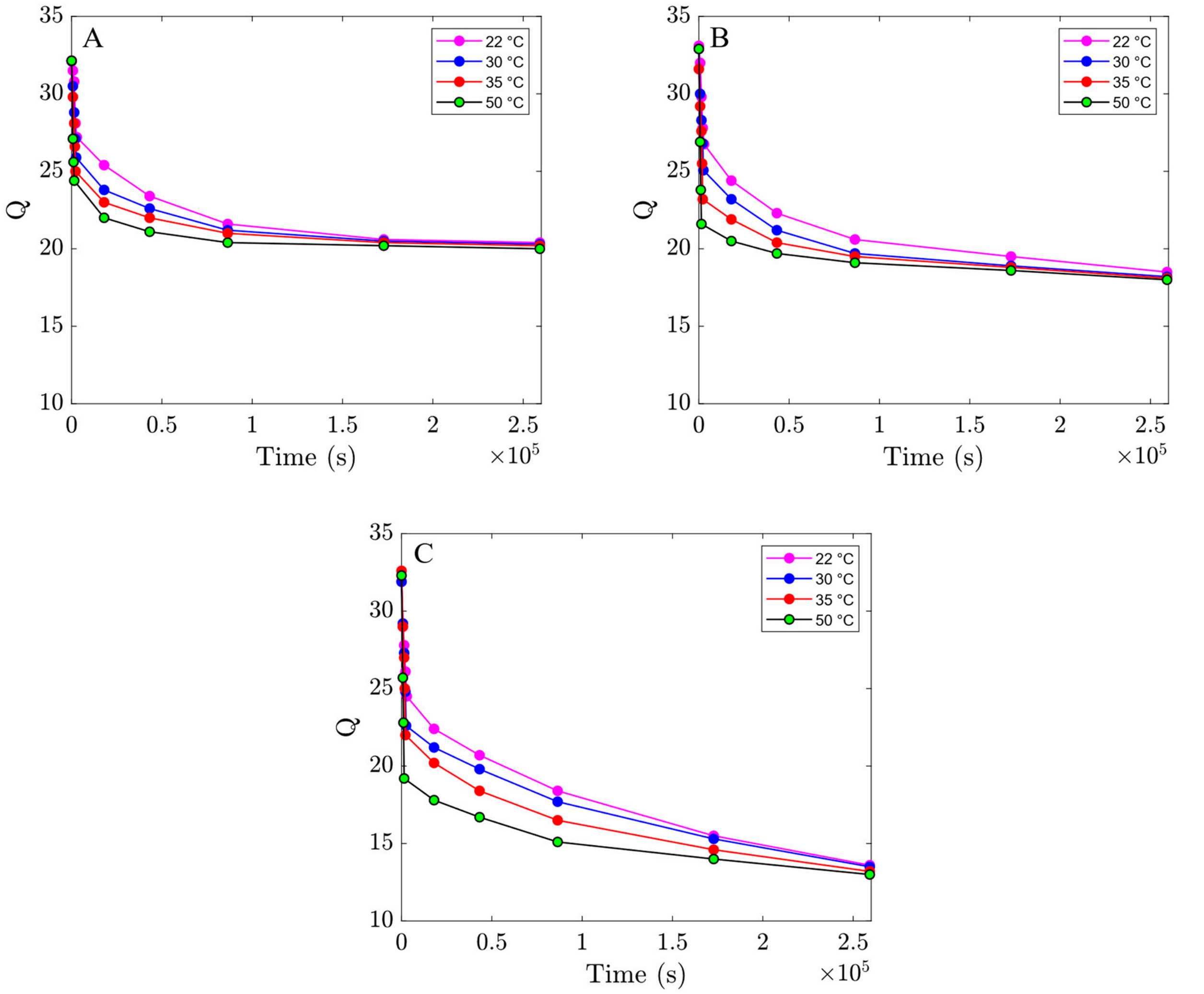

Figure S1: Degree of swelling Q versus time t for organohydrogels in pure (A) methanol, (B) ethanol and (C) 2-propanol at various temperatures T.
